# Chemically engineered unzipped multiwalled carbon nanotube and rGO nanohybrid for ultrasensitive picloram detection in rice water and soil samples

**DOI:** 10.1038/s41598-023-34536-7

**Published:** 2023-06-19

**Authors:** Daphika S. Dkhar, Rohini Kumari, Pranjal Chandra

**Affiliations:** Laboratory of Bio-Physio Sensors and Nano-Bioengineering, School of Biochemical Engineering, Indian Institute of Technology (BHU) Varanasi, Varanasi, Uttar Pradesh 221005 India

**Keywords:** Environmental impact, Graphene, Nanoscale materials

## Abstract

Picloram (4-Amino-3,5,6-trichloro pyridine-2-carboxylic acid) is a chlorinated herbicide that has been discovered to be tenacious and relatively durable in both soil and water. It is known to have adverse and unpleasant effects on humans causing several health complications. Therefore, the determination of picloram is profoundly effective because of its bio-accumulative and persistent nature. Because of this, a sensitive, rapid, and robust detection system is essential to detect traces of this molecule. In this study, we have constructed a novel nanohybrid system comprising of an UZMWCNT and rGO decorated on AuNPs modified glassy carbon electrode (UZMWCNT + rGO/AuNPs/GCE). The synthesized nanomaterials and the developed system were characterized using techniques such as SEM, XRD, SWV, LSV, EIS, and chronoamperometry. The engineered sensor surface showed a broad linear range of 5 × 10^–2^ nM to 6 × 10^5^ nM , a low limit of detection (LOD) of 2.31 ± 0.02 (RSD < 4.1%) pM and a limit of quantification (LOQ) of 7.63 ± 0.03 pM. The response time was recorded to be 0.2 s, and the efficacy of the proposed sensor system was studied using rice water and soil samples collected from the agricultural field post filtration. The calculated recovery % for picloram in rice water was found to be 88.58%—96.70% (RSD < 3.5%, n = 3) and for soil it was found to be 89.57%—93.24% (RSD < 3.5%, n = 3). In addition, the SWV responses of both the real samples have been performed and a linear plot have been obtained with a correlation coefficient of 0.97 and 0.96 for rice and soil samples, respectively. The interference studies due to the coexisting molecules that may be present in the samples have been found to be negligible. Also, the designed sensor has been evaluated for stability and found to be highly reproducible and stable towards picloram detection.

## Introduction

Chlorinated pesticides have been widely used to deter and control manifestations and plant diseases in agricultural premises for over 50 years. They are anthropogenic environmental contaminants that have been linked to probable or well-known endocrine diseases as a result of chronic or widespread toxicity^1^. Picloram is often toxic to living beings even at low-dose exposures^2^. Moreover, these pesticides are frequently transformed into the surface and substrate of the soil, as well as groundwater, and eventually enter the human body via food chains. Although the usage of such pesticides has been prohibited or restricted in many countries, some of them are nevertheless used regularly for weed and pest management^2^. Picloram is used to limit broad-leaved weeds in pasturages and grazing land, as well as wheat, woody plants, oats, and barley^3,4^. Considering that picloram has a potential to damage surface as well as groundwater supplies, the Environmental Protection Agency (EPA), United States labeled it a "restricted use" pesticide and made it mandatory to be used only by authorized personnel and individuals under their supervision^5^. This pesticide is exceedingly tenacious in soil, particularly in dry and cold areas, and even in the most ideal conditions, its degradation takes place gradually via bacteria^6^. Its half-life in the soil is reported to be in the range of 1 to 13 months based on certain circumstances^7^. At a concentration of 470 g/mL, it can easily seep into the soil and groundwater by virtue of agricultural usage and elimination of inappropriate wastes^8,9^ thus damaging adjacent non-target species of crops from purified ones and posing a hazardous danger to aquatic species^10–12^. Recent research has identified picloram as an ecological hormone of low toxicity but high carcinogenicity^13^. Human exposure to an elevated concentration of picloram can cause central nervous system disorders, weight loss, weakness, diarrhea and others^11^. The EPA prohibited the utilization of picloram and established its level in drinking water at 0.5 μg/mL to safeguard against several health complications^14^. In practical terms, sensitive detection of trace amounts of picloram is in high need for human health and environmental protection.

The most widely used conventional techniques for detecting and quantifying picloram are mass spectrometry/capillary electrophoresis and gas/liquid chromatography^15–17^. Though these methods are very sensitive and have lower LODs, they usually need expertise, lengthy sample preparation, purification processes, and sophisticated laboratory setups. Although radioimmunoassays (RIA) and enzyme-linked immunosorbent assays (ELISA) have shown to be efficient techniques for picloram detection, their use for on-site pesticide management is constrained by their complex washing procedures, lengthy incubation times, radiation risks, and huge instruments^18,19^. Therefore, a more practical, simple, and cost-effective approach is required. One such approach is the potent electrochemical studies of diverse molecules which is on the rise in the fields of clinical, biomedical, and environment^20,21^. Such methods have beneficial qualities, such as easy fabrication, minimal cost, quick response time, simple procedures, high selectivity, accuracy and excellent sensitivity in miniaturized settings^22–24^. The electrode's electroanalytical action is prompted by its working surface area and capacity to transmit electrons. Therefore, one effective way for improving the electrochemical efficacy of the electrode is to develop distinct and effective composite systems^25,26^. This can be accomplished by modifying and fabricating electrically conductive and catalytic nanomaterials to the electrode surface including nano-structures, metal oxides, conducting polymers, among others^27–30^. Electrochemical transduction-based biosensor devices in the environmental field are to deliver a quick, on-site, and completely automated detection approach of pollutants requiring no sample pre-treatment methods. Due to the above mentioned advantages, these devices have lately gained popularity as a result of their improved sensitivity, quick method of detection, and can be downsized for point-of-care analysis^31^. Very few electrochemical studies have been performed to detect picloram. Regarding the electrochemical detection of picloram as a target molecule, Mutharani et al. designed a voltametric sensor based on a thermo-responsive PVCL-tethered MWCNT using DPV^14^. Similarly, Bandzuchová et al. constructed a diamond film electrode doped with boron for picloram detection using DPV^12^. A few other sensors including immunosensors and electrochemical sensors have been constructed for the detection of picloram^32,33^. As compared to the previously proposed works, our system is deployable as it does not involve tedious setups, or complex sample preparation and shows an excellent response time. Most importantly, the detection range of the sensing system also covers the minimal level of picloram permitted by the EPA in drinking water. Hence, a novel composite system comprising materials that can detect picloram in low detection limit in complex matrices with fast response time is much needed for on-site surveillance.

Two-dimensional nanomaterials are known to have prominent features for sensing purposes such as high electrical conductivity, unique optical properties, high mechanical stability, flexibility, etc^34,35^ Nevertheless, MWCNTs have distinctive characteristics and are frequently explored for use in fuel cells, batteries, supercapacitors, biosensors, among others^36^. The development of biosensors has made use of MWCNTs as electrode materials by virtue of its characteristics such as large surface area, exceptional electrical conductivity, high corrosion resistance, and distinctive pore structure^37,38^. The potential of MWCNTs to store charges through the formation of electric double layers across the interface between the electrolytes and the electrode has been demonstrated to be electrostatic in nature^39^. However, UZMWCNT are a newly developed material created from the precursor MWCNTs. Due to its distinctive electrical, thermal, optical, magnetic, doping, and mechanical characteristics, it has drawn a great deal of attention from both theoretical and experimental researchers^40,41^. Unzipped graphene-like carbon nanotubes feature an elongated shape with a significant edge density, enhanced aspect ratio, and defects sites, along with high-quality graphene nanosheets. Such distinguishing characteristics render them a great contender for electrochemical-based sensors with promiscuous and enhanced current responses as well as reduced LODs for target analytes^42^. MWCNTs are wrapped into multiple graphene sheets hence, unzipping it results in a nanosheet-like structure with C–C bond and sp^2^ hybrid orbitals^42^. Since on-site and rapid detection of picloram by electrochemical transduction is solely dependent on the electrocatalytic activity of the sensing probe, it is distinct to design and develop a highly sensitive sensing system based on several nanomaterials. Several researchers have reported reduced graphene oxide (rGO) to be conductive and the recently synthesized unzipped multiwalled carbon nanotube (UZMWCNT) has also shown promiscuous electrical conductivity and electron transfer properties^42–44^. Therefore, we hypothesize that a nanohybrid system comprising of both may showed an enhanced electron transfer property that may be attributable to the integration and synergistic effect of rGO with the UZMWCNT. This nanohybrid system has not been reported so far and we believe that the nanohybrid may form a layered structure modified with AuNPs which may offer a stable electrode surface. Square wave voltammetry (SWV) has been extensively employed in the characterization and evaluation of the electrochemical performance of sensors and biosensors over the years. It is one of the sensitive electrochemical techniques. SWV is frequently used because of its capacity to operate at high frequencies. Comparatively to other pulse techniques, square wave studies may be conducted swiftly and can retain electroactive species at the electrode surface^45^.

In this work, a novel nanohybrid system comprising of the UZMWCNT and rGO was synthesized. The synthesized nanocomposite exhibits superior electrochemical properties as compared to UZMWCNT and rGO alone. The sensor construction comprises the nanohybrid synthesized i.e., UZMWCNT+rGO on a GCE modified with electrodeposited gold nanoparticles (AuNPs). The synthesized nanomaterials were characterized using scanning electron microscopy (SEM) and x-ray diffraction (XRD). This final sensor probe comprising the novel nanocomposite showed tremendous properties including an enhanced electrocatalytic response, good sensitivity and, high stability. The probe was then employed for the electrochemical detection of the herbicide, picloram. Throughout the experiment, electrochemical studies including linear sweep voltammetry (LSV), electrochemical impedance spectroscopy (EIS), square wave voltammetry (SWV), and chronoamperometry were conducted for assessment of the overall sensor’s performance including LOD, LOQ and linear range. The sensor was finally tested in rice water and soil sample to assess its feasibility in environmental monitoring of picloram. In addition, another parameter assessed was the response time of the sensor developed and it was found out to be 0.2s.

## Materials and methods

### Chemicals and instrument

The experiment conducted made use of analytical-grade chemicals. Chloroauric acid (HAuCl_4_,) ultrapure 99.99%, MWCNT extrapure 95%, and graphite synthetic powder (Type IV) pure were purchased from SRL Pvt. Ltd. Sodium biphosphate (Na_2_HPO_4_) extra pure 99% and Sodium monophosphate (NaH_2_PO_4_) extra pure AR 99% were obtained from SRL. Ltd. Picloram, extra pure 95% was also obtained from SRL Pvt. Ltd. Hexaammineruthenium (III) chloride 98% pure and Nafion^®^ 117 were purchased from Sigma-Aldrich Chemical Co. All of the electrochemical analyses were carried out using an electrochemical workstation (Palm sense 4.0) with silver/silver chloride (Ag/AgCl) (saturated with KCl, as a reference electrode), platinum wire (Pt, as an auxiliary electrode) and GCE (as a working electrode).

### Chemical unzipping of MWCNT

UZMWCNT was synthesized by a modified chemical unzipping process^46^. Briefly, 250 mg of MWCNT was added to a beaker containing 50 mL of concentrated H_2_SO_4_ and stirred for 24 hrs. After the 24 hrs period, 1.25 g of KMnO_4_ was added to the slurry and agitated in RT for 1 hr. The solution was allowed to stir for another 1 hr at 65 °C after which it was allowed to cool at RT. The mixture was then poured into 250 mL chilled deionized water containing 30% H_2_O_2_ (25 mL). The slurry was further centrifuged at 5000 RPM several times washing with 20% HCl and deionized water. The resultant pellet was eventually dried in an oven at 60 °C in order to obtain a powdered form. The prepared samples were kept in a vacuum desiccator and were used for sensor fabrication without further fabrication.

### Synthesis method of reduced graphene oxide

Graphene oxide (GO) was used as a starting material to synthesize reduced graphene oxide (rGO). Initially, GO was prepared using the well-known Hummer’s synthesis protocol^47^. Briefly, 0.450 g of graphite powder was taken in a 60 mL solution prepared with concentrated H_3_PO_4_ and H_2_SO_4_ in a ratio of 1:9. by Next, 2.64 g of KMnO_4_ was added slowly to the solution. Further, the slurry was neutralized via the addition of 1.35 mL of 30% v/v H_2_O_2_ to the mixture and agitated for 10 mins. Next, 30% of HCl (10 mL) and 60 mL of deionized water were added to the mixture. The synthesized GO was extracted by centrifuging it at 5000 RPM and left to dry at 70 °C overnight to obtain a powdered form. Next, 0.04 g of the powdered GO was added to a 40 mL mixture of DMF: deionized water (9:1) to form a colloidal solution. The mixture was sonicated for 2 hrs followed by a dropwise addition of hydrazine hydrate at 47 °C and thereafter heated for 24 hrs at 100 °C. The final solution was subjected to washing using ethanol and deionized water repeatedly till the pH dropped to near neutral. The material was finally allowed to dry in a hot air oven at 30 °C till a powdered form is obtained.

### Preparation of working solutions

For the electrochemical study of the various modified surfaces, 5 mM Phosphate buffer saline (PBS) of pH 7.0 and 5mM ruthenium hexamine (Ruhex) of pH 7.0 as a supporting electrolyte were prepared. For real sample analysis of picloram in rice water and soil samples, 0.1 M PBS (pH 7.0) was prepared and used throughout the experiment. The different concentrations of the analyte and interfering molecules were prepared using 0.1 M PBS.

### Fabrication of UZMWCNT + rGO/AuNPs/GCE sensing probe

**Figure 1:**
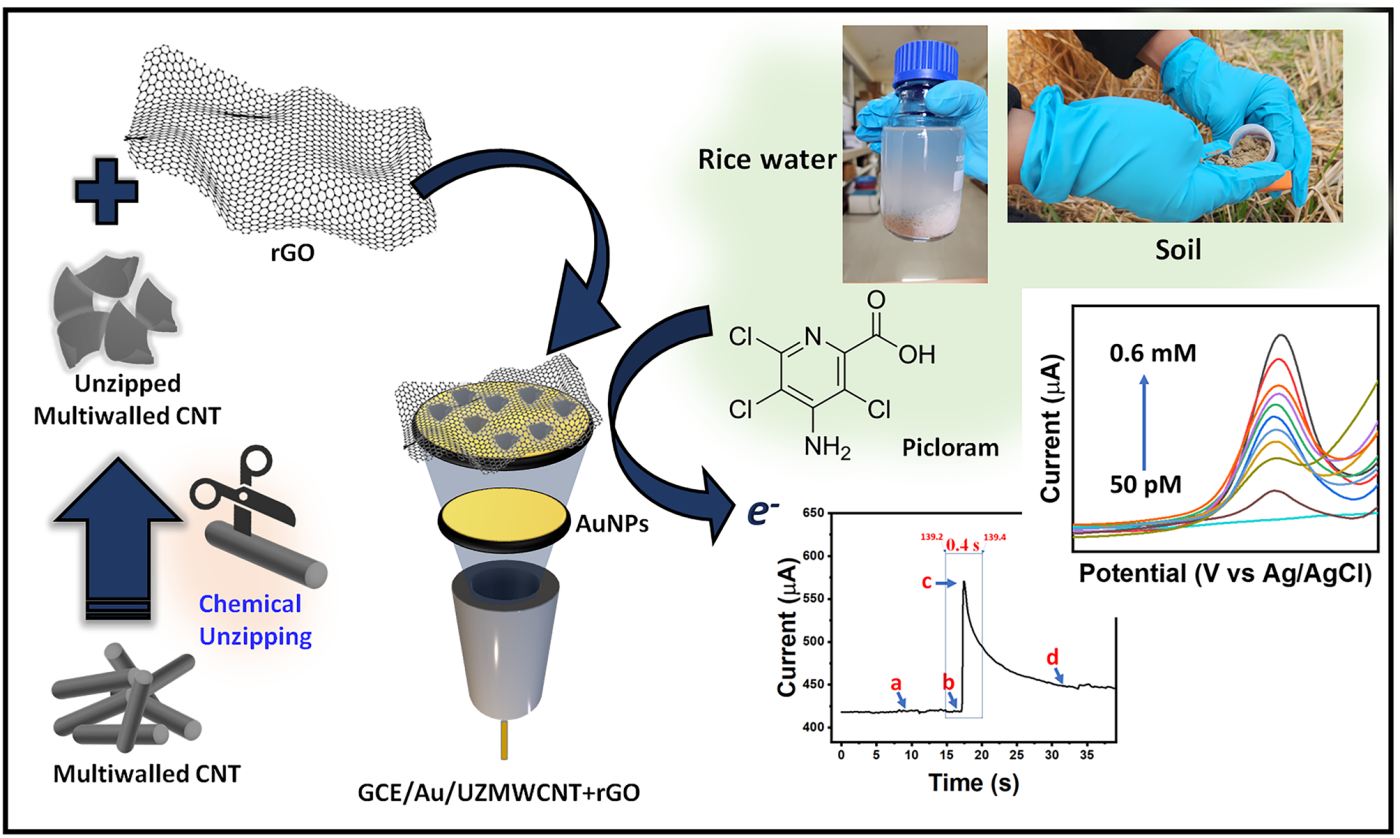
Schematic representation of the fabrication of the sensor and its analytical performance and response time for picloram detection.

At the first stage, a nanohybrid solution (1 mg/mL) of UZMWCNT and rGO was prepared by initially dissolving each of the powdered forms of UZMWCNT and rGO in MilliQ water in a ratio of 1:1 and sonicated for 1 min to obtain a homogenous mixture. 2 μl of 0.02% Nafion was added to the mixture prior to fabrication. The sensor probe was engineered by sequentially modifying the surface of a GCE as shown in Fig. 1. The GCE was pre-cleaned by polishing the electrode thoroughly on a polishing pad containing alumina slurry and further washed with deionized water. Initially, the working electrode was modified with electrodeposited AuNPs (acidic, 0.5 M H_2_SO_4_ containing 0.0025% HAuCl_4_) utilizing a potential step approach by conducting LSV between +1.5 and +0.4 (V) vs. Ag/AgCl. The following are the AuNPs electrodeposition conditions: three sweeps at 0.1 V/s scan rate and a deposition time of 60 seconds. Prior to fabrication, the AuNPs were sonicated for 10 minutes to prevent agglomeration of the nanoparticles. From the as-prepared nanocomposite solution of UZMWCNT+rGO, 10 μl was systematically layered onto the electrode modified with AuNPs (AuNPs/GCE). The final sensing probe termed UZMWCNT+rGO/AuNPs/GCE was used for the rapid and simple electrochemical detection of picloram in real samples as shown in Fig. 1.

### Real sample preparation

Validation of the designed probe UZMWCNT+rGO/AuNPs/GCE was carried out using rice water and soil as real sample matrices due to the fact that picloram is readily present in rice and agricultural soil^48^. The samples were collected from the agricultural field of Banaras Hindu University, Varanasi. The spike/recovery testing model was employed to study and evaluate the potential and ability of the designed sensor toward picloram detection. The current responses of the two samples were assessed and a comparison study was performed with that of the standard calibration obtained from picloram solution in a standard buffer.

### Ethics approval

Ethics approval was not required for this research.

### Consent to participate

All the authors participated in this article.

## Results and discussions

### Characterization of the sensing probe

**Figure 2:**
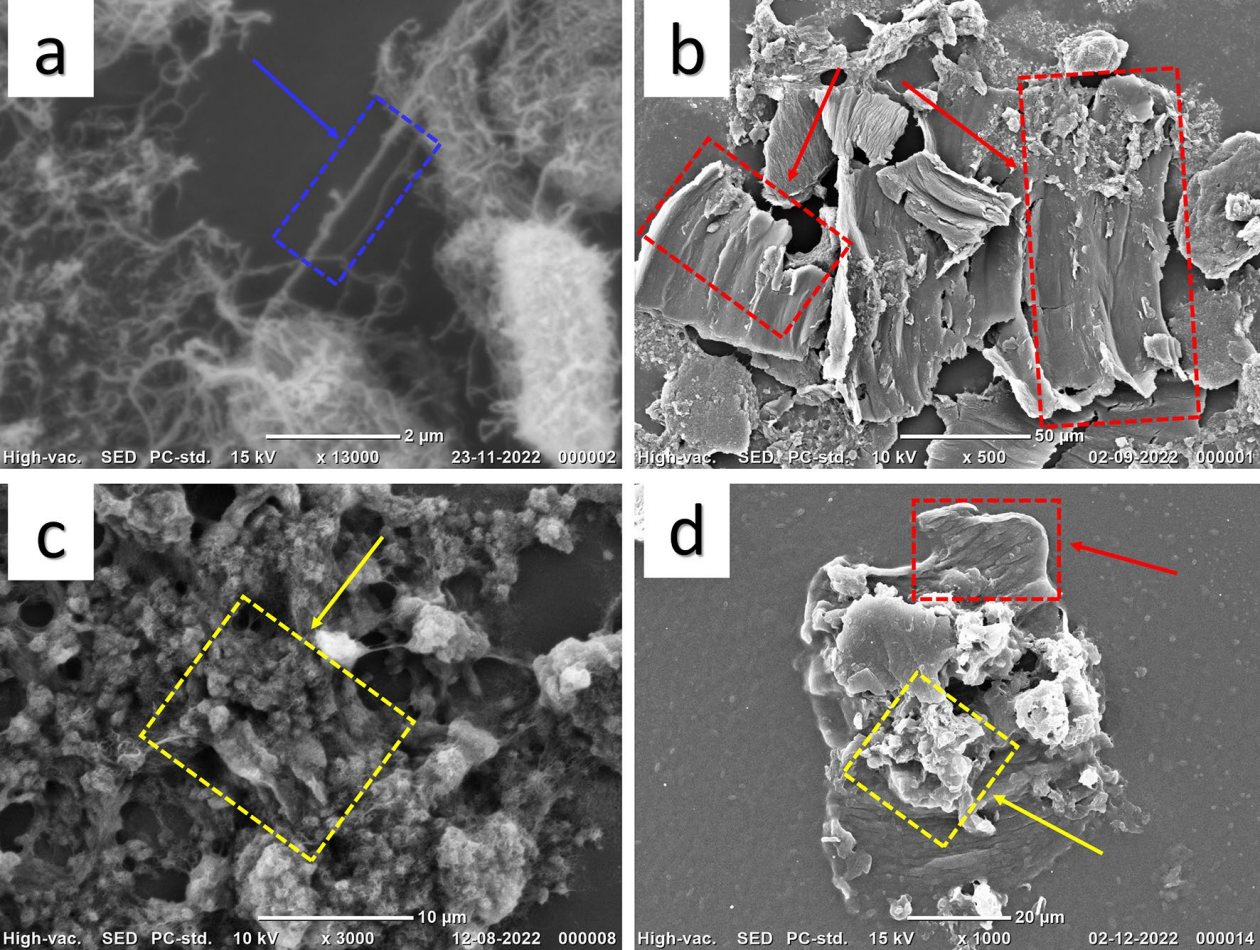
SEM images of the nanomaterials synthesized (**a**) multiwalled carbon nanotube showing tube-like structure (**b**) unzipped multiwalled carbon nanotube surface morphology with graphene-like nanosheets (**c**) reduced graphene oxide (**d**) nanocomposite comprising of multiwalled carbon nanotube and reduced graphene oxide.

**Figure 3:**
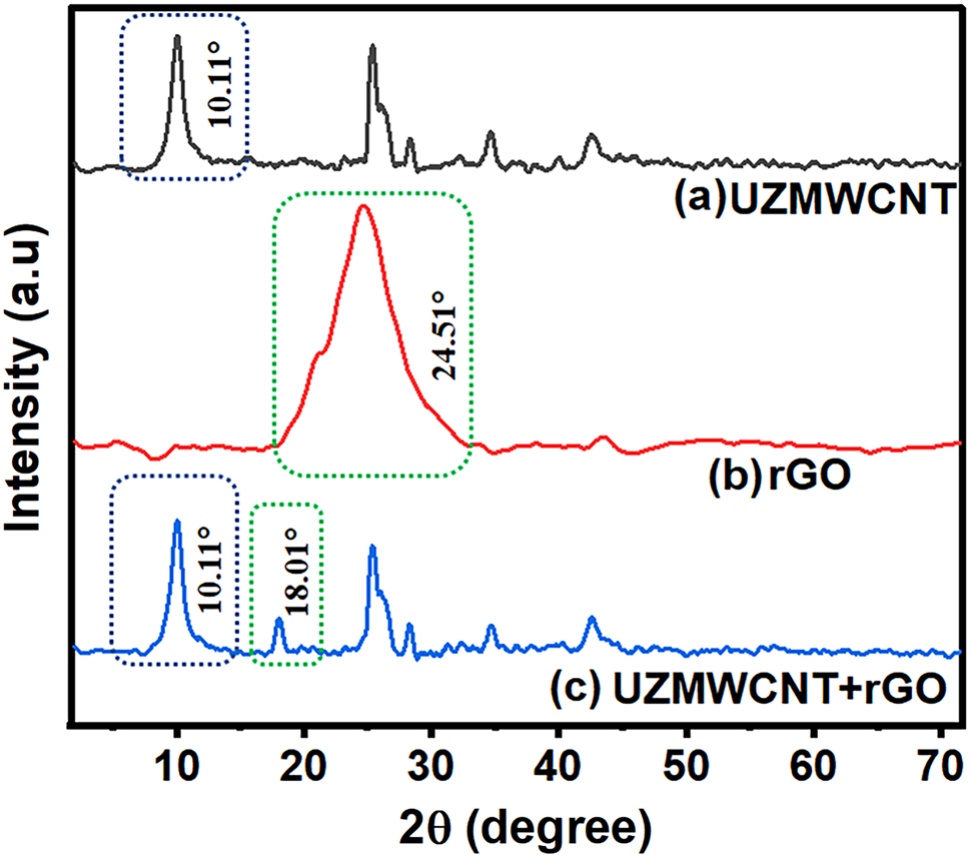
XRD patterns obtained for (**a**) UZMWCNT (**b**) rGO (**c**) UZMWCNT + rGO.

In the first stage, we characterized the nanohybrid materials that have been utilized to fabricate the sensor probe. For the synthesis and morphology of UZMWCNT and rGO, we have characterized the nanomaterials using SEM. The SEM image of the precursor MWCNT as shown in Fig. 2a **(**blue box**)** displays the tube-like structure and Fig. 2b **(**red box**)** reveals the unzipped structure of the tube-like MWCNT. This demonstrates the successful synthesis of the UZMWCNT using the chemical unzipping method. Further, we have also performed SEM to reveal the structure of reduced graphene oxide (yellow box) (Fig. 2c) and the presence of both UZMWCNT and rGO in the nanohybrid synthesized was confirmed (yellow and red boxes) (Fig. 2d). Also, to determine the crystallinity and composite formation of UZMWCNT, rGO and the nanohybrid UZMWCNT+rGO, XRD study was performed. The XRD examination was carried out at 2 Bragg's angle, with a scan rate of 20° per minute, step-size 0.02 and a range of 5° to 80°. 40 mg of UZMWCNT, rGO and a mixture of both in powdered form was used to check the diffraction pattern. Figure 3 shows the XRD pattern of the as prepared nanomaterials and the nanohybrid. The XRD pattern of UZMWCNT revealed a characteristic peak at 2θ=10.11º corresponding to the (002) interlayer spacing (Fig. 3a), which is consistent with the earlier reported study for UZMWCNT^49^. This indicated the succesful synthesis of UZMWCNT to graphene-like sheets. In addition, there are several other peaks observed at 2θ=25.37º, 2θ=28.27º, 2θ=34.75º and 2θ=42.55º. These additional peaks may be attributable to the residual structure of MWCNTs that may not have been fully exfoliated^49^. For the XRD pattern of rGO, a characteristic peak was observed (Fig. 3b) at 2θ=24.51º corresponding to the (002) interlayer spacing^50^. Similarly, the formation of the nanohybrid comprising of UZMWCNT+rGO was observed in the diffractogram shown in Fig. 3c. In this case, the peaks observed in (a) were also present with an additional peak at 2θ=18.01º. In the nanohybrid system, the peak at 2θ=18.01º may correspond to rGO which was observed to be shifted from 2θ=24.51º to 2θ=18.01º. Since the nanohybrid comprises of two carbon sources, the shift observed may be a result of an increased synergistic effect possibly due to the C=O interaction as reported in a previous study^21^. Next, utilizing the nanohybrid of rGO and UZMWCNT the sensor probe was further fabricated. Since we intended to perform an electrochemical sensor for picloram detection, we further investigated the electrochemical/catalytic performance of each layer of the sensor with adequate controls.

**Figure 4:**
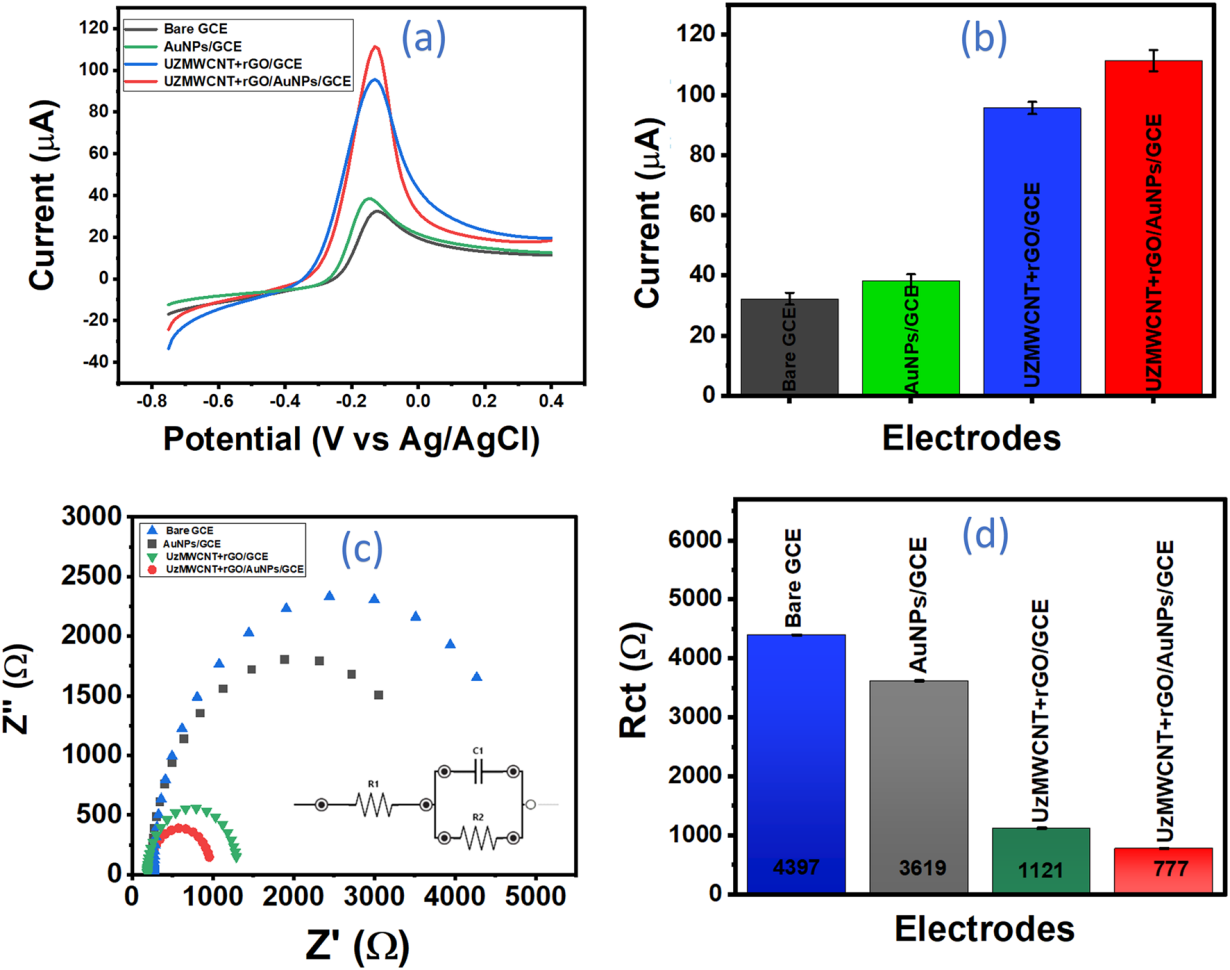
Electrochemical studies of the different electrode modifications at every stage (**a**) LSV responses of bare GCE (black), AuNPs/GCE (green), UZMWCNT + rGO /GCE (blue), UZMWCNT + rGO/AuNPs/GCE (red) in 5 mM Ruhex solution (**b**) Comparative histogram of corresponding LSV peak responses: GCE (black), AuNPs/GCE (green), UZMWCNT + rGO /GCE (blue), UZMWCNT + rGO/AuNPs/GCE (red) (**c**) EIS study showing Nyquist plots of bare GCE (blue), AuNPs/GCE (grey), UZMWCNT + rGO/GCE (green) and UZMWCNT + rGO/AuNPs/GCE (red) in 5 mM PBS (**d**) Corresponding histogram of Rct values of the electrodes.

For the purpose of the study of electron transfer properties including catalytic and conductivity of the as-synthesized nanohybrid, the fabricated sensor probe UZMWCNT+rGO/AuNPs/GCE was extensively characterized using electrochemical techniques. The electrodeposition of AuNPs on the electrode surface was validated as reported in previous studies^35^ wherein a prominent reduction peak was detected at 0.9V vs. Ag/AgCl, owing to the reduction of Au^3+^ to Au^0^ on the electrode's surface, resulting in the formation of AuNPs. For every step of the electrode study, the LSV approach was utilized using a solution containing Ruhex to evaluate all of the modified electrodes, and the anodic peak outputs were logged at a potential range of −0.75 to +0.4 V at a scan rate of 50 mV/s. The respective LSVs of bare GCE (black), AuNPs/GCE (green), UZMWCNT+rGO/GCE (blue), and the final sensor probe UZMWCNT+rGO/AuNPs/GCE are shown in Fig. 4a. An increase in the current outputs due to Ruhex was observed in AuNPs/GCE as compared to bare GCE as the latter possesses a sluggish electron transfer behavior. In the next modified surface with UZMWCNT+rGO/GCE, a further increment of current was observed. This could be by virtue of an increased electrocatalytic and electron transfer properties of the nanohybrid. However, the final sensing surface modified with AuNPs and UZMWCNT+rGO (UZMWCNT+rGO/AuNPs/GCE) showed a maximum amplified current peak response compared to the previously tested surfaces. The final modified electrode showed a commendable and fascinating enhancement of current responses most likely due to the synergistic and combined effect of the synthesized nanohybrid with AuNPs. Thus, it can be speculated that the highest current observed in the developed electrode UZMWCNT+rGO/AuNPs/GCE may be due to its highest conducting nature and capability of assisting electrochemical signal effectively. Therefore, the sensor probe UZMWCNT+rGO/AuNPs/GCE was used for further analysis and detection of picloram. The peak responses of the different modified electrode surfaces have also been represented in a comparative histogram (Fig. 4b). In addition, we have also calculated the effective electrode surface areas (A) of all the modified electrodes to evaluate and compare the electrocatalytic activities as well as the charge transfer properties using Randles Sevcik’s model (Eq. 1)

1 $$I_{p} = 2.69 \times10^{5} n^{3/2} ACD^{1/2} v^{1/2}$$

wherein, I_p_: peak current (amperes); n: number of electrons transferred in a redox reaction [in this work, n=1]; A: effective surface area (cm^2^); C: concentration of the electroactive species (mole/cm^3^); D: diffusion coefficient (cm^2^/s) [for Ruhex= 0.53×10^5^]; v: scan rate (V/s)

**Figure 5:**
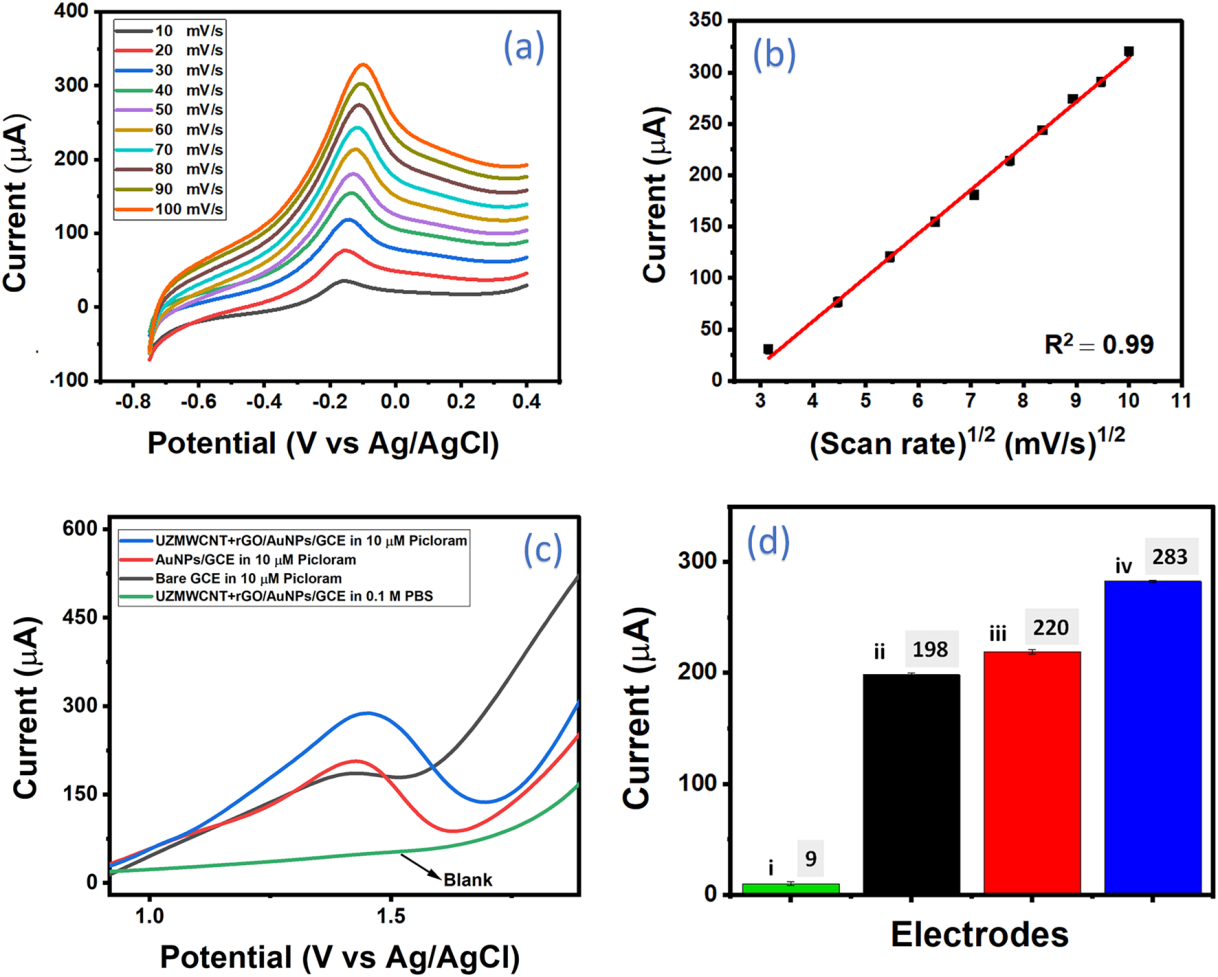
(**a**) Scan rate dependent study of the UZMWCNT + rGO/AuNPs/GCE sensing platform in 5 mM Ruhex ranging from 10 mV/s to 100 mV/s (**b**) Corresponding anodic peak currents showing linearity with R^2^ of 0.99 (**c**) Study of response currents of stages of fabrication of electrodes with respect to picloram (10 μM) using SWV showing blank (green), bare GCE (black), AuNPs/GCE (red) and UZMWCNT + rGO/AuNPs/GCE (blue) (**d**) Histogram showing corresponding current values of the electrodes with respect to picloram (10 μM) detection: (i) UZMWCNT + rGO/AuNPs/GCE [blank (no picloram)] (ii) Bare GCE (iii) AuNPs/GCE (iv) UZMWCNT+rGO/AuNPs/GCE in 10 µM picloram.

The effective surface area of all the fabricated electrodes GCE, AuNPs/GCE, GCE/UZMWCNT+rGO, and UZMWCNT+rGO/AuNPs/GCE was calculated to be 2.04 cm^2^, 2.47 cm^2^, 6.08 cm^2,^ and 7.21 cm^2^ respectively. The results indicated that the effective surface area of the final surface UZMWCNT+rGO/AuNPs/GCE is much higher than that of GCE, AuNPs/GCE, and GCE/UZMWCNT+rGO. To support and complement the findings of LSV in terms of higher current magnitude, we have validated the results by performing EIS in 0.5 mM PBS where the resistance of the electrodes was examined. Figure 4c shows the Nyquist plots of the different modified surfaces and the EIS data was recorded at ten points per decade sampling rate with an open circuit voltage ranging from 10 to 104 Hz. The Rct values obtained by fitting the experimental data to the equivalent circuit (inset) was found to be 7518(±13.21) Ω for bare GCE, 7392 (±13.14) Ω for AuNPs/GCE, 1121.333 (±14.24) Ω for GCE/UZMWCNT+rGO and 508.7 (± 15.68) Ω for UZMWCNT/AuNPs/GCE (Fig. 4d). Interestingly, the lowest Rct was noted for the final sensing probe as compared to the other surfaces implying that the constructed probe has the most rapid mechanism of electron transfer at the electrode/electrolyte interface. Further to assess the mechanism of charge transfer as well as the stability of the final probe surface, we have conducted a scan rate study from 10 mV/s to 100 mV/s in 5 mM Ruhex prepared in 5 mM PBS Fig. 5a. The peak outputs were observed to be precisely proportional to the square root of the scan rates and a correlation coefficient of 0.99 was obtained as shown in Fig. 5b. (equation). This indicates a diffusion-controlled charge transfer behavior of the developed working electrode area which corresponds to a decrease in the diffusion layer as a result of faster scan rates hence an enhancement in the current responses was ascertained^35,51^. Thus, the voltametric and EIS studies complement each other precisely, indicating that the final probe facilitated an increase in electrical conductivity and finally assist in an enhancement of the signal response.

### Analytical performance of UZMWCNT + rGO/AuNPs/GCE sensor probe

**Figure 6:**
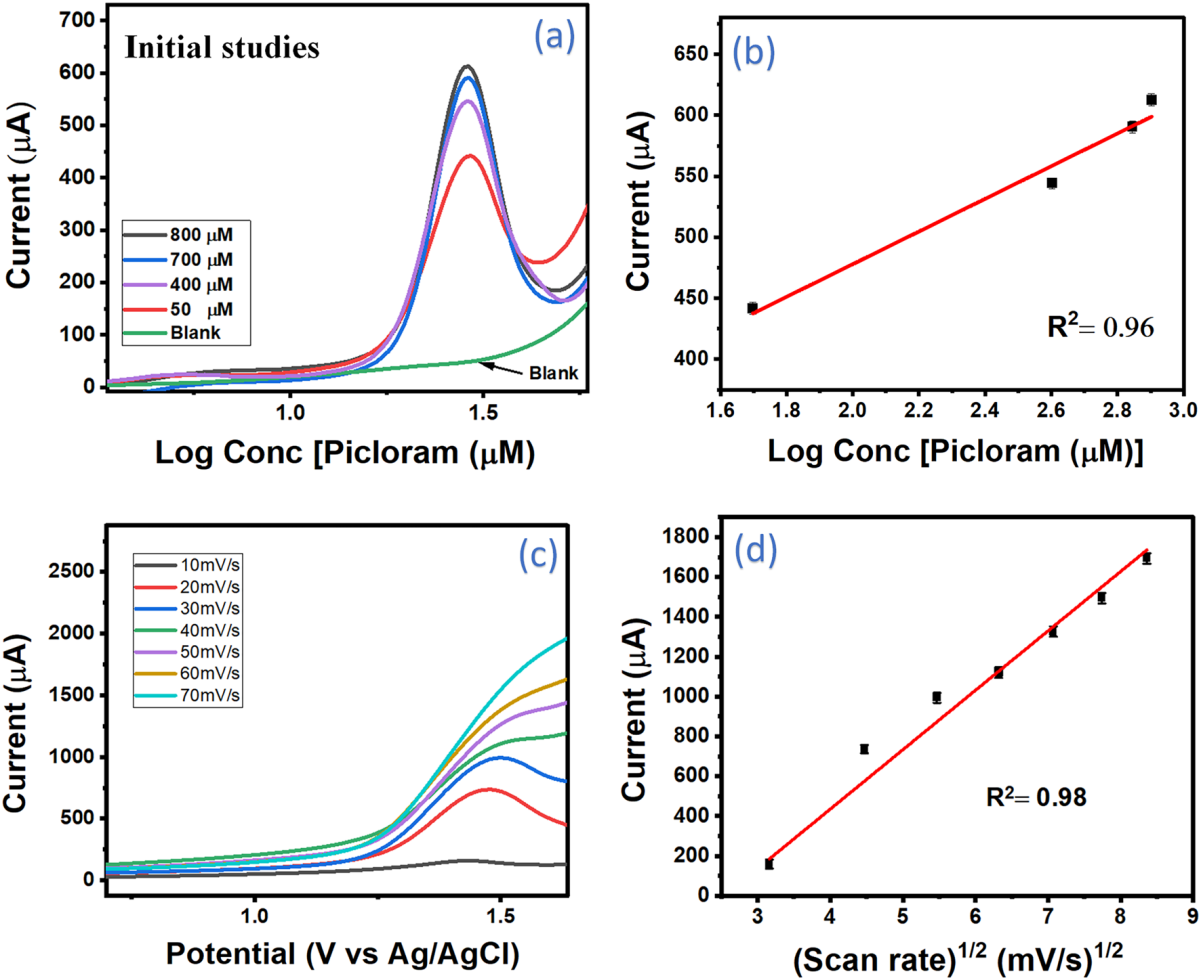
(**a**) Electrochemical study of concentration dependent detection of picloram using SWV showing blank, no picloram (green), 800 µM picloram (black), 700 µM picloram (blue), 400 µM picloram (purple) 50 µM picloram (red) (**b**) Corresponding regression plot with R^2^ of 0.96 (**c**) Scan rate of the final sensing platform ranging from 10 mV/s to 70 mV/s (**d**) Corresponding linear regression plot with R^2^ of 0.98.

Following the successful evaluation of the developed sensor probe UZMWCNT+rGO/AuNPs/GCE via the characterization techniques, we have subjected the probe towards the detection of picloram. To assess the suitability of the designed sensor for the target molecule detection, we have conducted a SWV study on each of the electrode surfaces (Fig. 5c). For that, a bare GCE was dipped in 0.1 M PBS (pH 7.0) containing no picloram (negative control), and a SWV was carried out by applying a potential between −0.8V and +2.0V [t equilibration: 10s; E step: 0.01; amplitude: 0.1; frequency: 20.0]. In this case, no peak was observed (green) for blank. Similarly, in the next step, a SWV response was recorded using a bare GCE which was scanned under the same potential window in an electrochemical cell containing PBS with 10 μM of picloram. A distinct peak that was observed at 1.43 V (black), indicated the existence of picloram in the electrolyte solution which could be due to the electro-oxidation of the amino group of picloram during SWV. In the following step, the AuNPs-modified GCE was evaluated in a similar way using the same solution and parameters. Here, an amplified current peak (red) with a slight shift in potential peak towards the positive direction was observed. This could be a result of the enhanced electrocatalytic property of the AuNPs present on the surface of the GCE. In the last step, the developed UZMWCNT+rGO/AuNPs/GCE probe was applied to the same set of the above-performed experimental conditions. A remarkably enhanced current response (blue) was detected as compared to the other modified electrodes thus, demonstrating the ability of the sensor probe toward sensitive detection of picloram. A comparative histogram of the current responses has also been illustrated in Fig. 5d. An increase in the effective surface area and an amplification in the conductivity may be the other factors attributable to the observed current response of the final sensor probe. In addition, to further authenticate that the peak observed at 1.4 V was solely due to the electro-oxidation of picloram on the designed sensor matrix, we have performed two distinct control studies. In the first one, we conducted a concentration-dependent study wherein four random concentrations of picloram (800 μM, 700 μM, 400 μM, and 50 μM) were carried out. Figure 6a depicts the SWV responses of the sensor towards picloram where an enhancement in current responses with an increase in its concentration was observed. A representation of the regression plot from the concentration-dependent study is shown in Fig. 6b with a linear regression equation of ΔI (μA) = 210.19 (±39.57) +133.87 (±15.46) Conc [Picloram (μM)] and a correlation coefficient of 0.96. In the next control test, we conducted a scan rate study on the final electrode. With increasing scan rates from 10 mV/s to 70 mV/s, the peak currents were observed to be directly proportional to the square root of the scan rates mainly due to the diffusion-controlled electro-oxidation of picloram as shown in Fig. 6c and a correlation coefficient of 0.98 was obtained (Fig. 6d). In addition to the diffusion-mediated process, it is also worth mentioning that the current responses for picloram at higher scan rates are linear in function, which depicts that the fabricated sensor is stable at even higher scan rates. Both the outcomes of the experiments confirmed that the designed probe could sensitively and accurately detect picloram.

**Figure 7:**
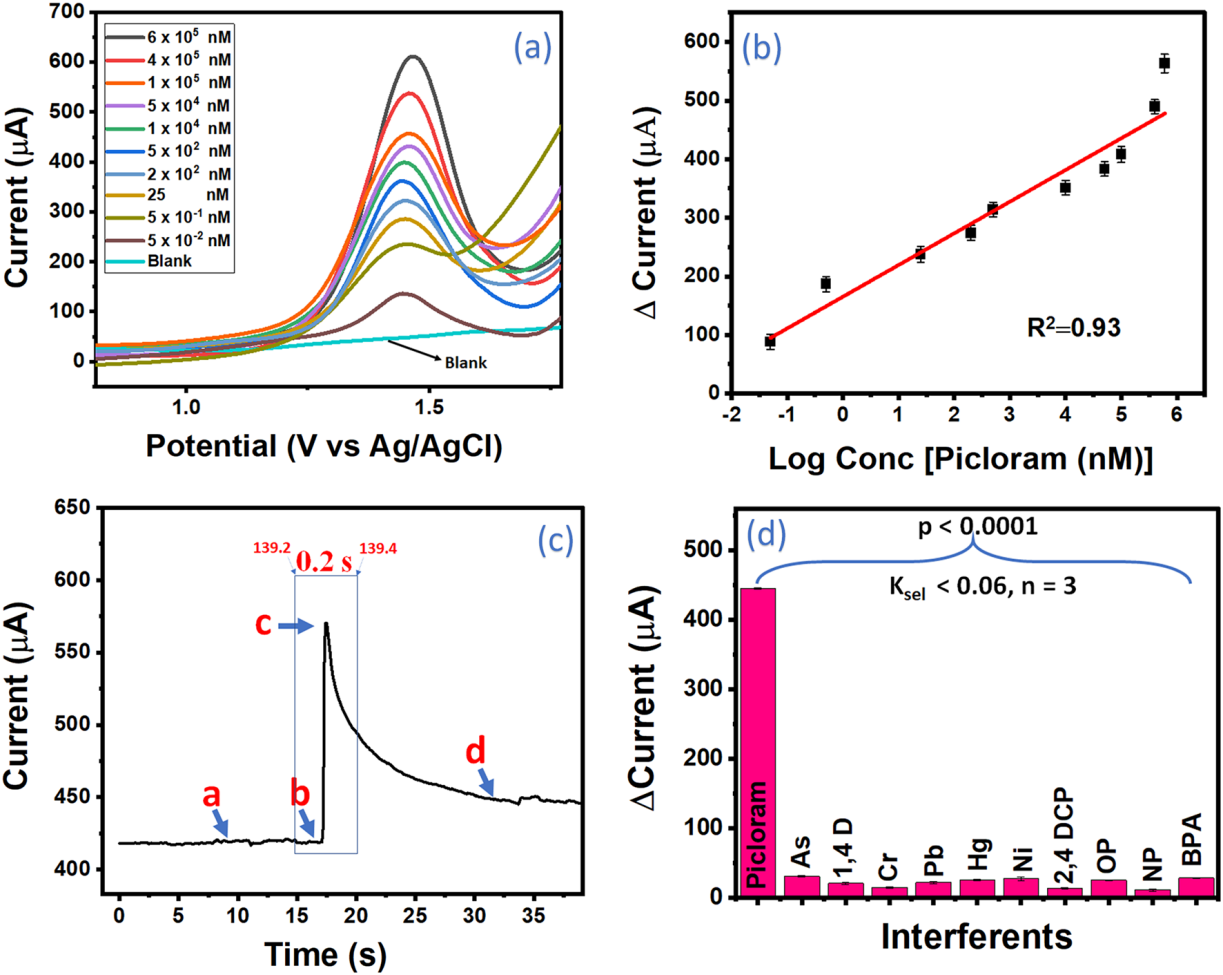
(**a**) SWV response of UZMWCNT + rGO/AuNPs/GCE sensing probe towards varying concentration of picloram ranging from 5 × 10^–2^ to 6 × 10^5^ nM as well as blank (no picloram) (**b**) Corresponding calibration plot showing linearity with R^2^ of 0.93 (**c**) Chronoamperometric study of the final probe in PBS injected with 0.4 mM picloram showing a response time of 0.2 s (**d**) Interference study of the UZMWCNT + rGO/AuNPs/GCE probe showing its highly selectivity towards picloram.

For further analysis of the analytical performance of the designed sensor UZMWCNT+rGO/AuNPs/GCE, we have evaluated it by assessing and quantifying varying concentrations of picloram via SWV in the range of −0.8V to +2.0V. Initially, to nullify the chances of the presence of impurities in the solution, several blank responses were recorded on the final probe under the same operational window to establish the baseline of the electrode. Afterward, a dose-dependent study of the same was recorded on the designed sensor probe. Figure 7a depicts the SWV responses of the calibration curve wherein a rise in the peak current was observed with an increase in the concentrations of picloram at a potential around 1.4 V vs Ag/AgCl. Based on SWV responses, a calibration curve was generated with a linear regression equation of ΔI (μA) =165.33 (±18.16) + 54.08 (± 4.87) Log conc [Picloram(nM)] and a correlation coefficient of 0.93 (n=3) (Fig. 7b).

Using the following equation, a LOD for picloram using the designed sensor probe was calculated:

2 $$\begin{aligned} LOD & = \frac{{3SD_{B} }}{Slope} = \frac{{3SD_{B} }}{{\frac{dy}{{dx}}}} = \frac{{3SD_{B} }}{{\frac{dy}{{d\ln x}} \times \frac{d\ln x}{{dx}}}} = \frac{{3SD_{B} }}{{\frac{dy}{{2.303\left( {d\log x} \right)}} \times \frac{1}{x}}} \\ & = \frac{{3\left( {2.303} \right)SD_{B} x}}{{\frac{dy}{{\left( {d\log x} \right)}}}} = \frac{{3\left( {2.303} \right)SD_{B} x}}{{\text{the slope of the semilog plot}}} \\ \end{aligned}$$

where SD_B_ is the blank’s standard deviation; x is the limit of quantification or lowest concentration detected.

Using Eq. (2), a LOD of 2.31 (± 0.02) (RSD <4.1%) pM and a broad linear range of 5×10^-2^ nM to 6×10^5^ nM was obtained. In addition, a limit of quantification (LOQ) of 7.63±0.03 pM was attained. To further add to the efficiency of the designed sensor probe, a response time was recorded using chronoamperometry in PBS at a constant potential of 1.4 V. Figure 7c depicts the chronoamperometric response to picloram addition on a stable current (a). After picloram injection (b), a substantial rise in the current response in current was observed between 139.2s and 139.4s (c, inset). After saturation of the signal, the current value exhibited a stable and significantly higher current value (d). The chronoamperogram clearly suggests that our sensor is very robust and is able to detect picloram in 0.2s. In this work, the rapid response time may be attributable due to the highly conducting and electrocatalytic nanohybrid system that assisted in the fast signal generation compared to the usual bioreceptor-based sensors such as an antibody, aptamer which requires an inherent complexation time(Suman et al. 2021). A comparative table of the previously reported sensors and our work has been shown in Table 1. As compared to the earlier reported studies as given in Table 1, our sensor platform achieved a lower LOD, broader linear range, and a rapid response time. Notably, our sensor is deployable as it is highly stable and is simple to manufacture. It is also worth mentioning that the detection strategy does not require any biorecognition element or any tedious fabrication steps that may limit with the development and implementation of this low-cost sensor system for picloram detection.

**Table 1:** A comparison of the previously developed systems and the present work for picloram detection (NR-not reported) wherein the lowest LOD was obtained in our wok.

Sl. No	Composite system	Detection technique	Linear range	Limit of detection	Real sample	Response time	References
1	Au@MWCNT-PVCL/ GCE	DPV	0.02–183 μM	1500 pM	Rice River water Soil	NR	^14^
2	MWCNT/Cr-MOF/ GCE	SWV	0.1–12.5 μM and 12.4–40 μM	60,000 pM	River Water	NR	^32^
3	Anti-PCR/Chitosan/ AuNPs/GCE	CV	0.005 to 10 µg/mL	20.7 pM	Rice, Lettuce, Paddy field water	NR	^33^
4	Boron-doped diamond film electrode	DPV	0.8 to 48.07 μmol^−1^ L	7.0 × 10^4^ pM	Tap water, natural water	NR	^12^
5	UZMWCNT + rGO/AuNPs/GCE	SWV	5 × 10^–2^ to 6 × 10^5^ nM	2.31 ± 0.02 pM	Soil, Rice	0.4 s	This work

Next, the interference caused by numerous compounds that may coexist with the constructed sensor in generation of signal has been investigated.

### Selectivity study

A sensor must be selective to its analyte in order to have commercial utility, and its reaction to interfering compounds must be evaluated^52^. In this experiment, the selectivity of the sensing probe towards picloram detection was performed in addition to the other possible interferents was also examined. The co-interfering molecules/xenobiotics such as mercury (Hg), lead (Pb), nickel (Ni), arsenic (As), chromium (Cr), 1,4-Dioxane (1,4 D), 2,4-Dichlorophenol (2,4 D), 4-octylphenol (OP), nonylphenol (NP), bisphenol A (BPA) which may be present in the soil^53–58^ were taken at a two-fold higher concentration to show the accuracy of our sensor. Under the same operational window using SWV, no peaks were observed at the vicinity of oxidation potential of picloram and the insignificant current outputs are shown in the histogram depicted in Fig. 7d. These results interpret that these molecules do not hinder the detection of picloram.

Moreover, the sensor's selectivity was inferred statistically inferred by calculating the coefficient of selectivity (*k*_*sel*_) using Eq. (3).

3 $$k_{sel} = \frac{{\left( {Signal} \right)interferent{ }}}{{\left( {Signal} \right)picloram}}$$

where k_sel_ denotes the coefficient of selectivity, (Signal)_interferent_ is the current response signal of the interfering molecules, and (Signal)_picloram_ is the current response signal of the sensor towards picloram.

Notably, the interfering molecules displayed no electrochemical response hence the sensor probe is found to be selective towards picloram. The obtained *k*_*sel*_ coefficient value for interferents was extremely low (*k*_*sel*_ < 0.06), indicating the designed sensor’s ability to detect picloram sensitively and Table 2 shows the* k*_*sel*_ values of the interferents. Further, the selectivity data is treated statically by conducting T-tests and determining the p-values for all the interferents against picloram and the results were found to be insignificant (p << 0.0001, n =3).

**Table 2:** K_sel_ values of picloram as well as that of the interfering molecules with the ΔCurrent values of each.

Sl No	Interfering molecules	ΔCurrent response at 1.43 V vs. Ag/AgCl	K_Sel_
1	Picloram	445.17 (± 5.78)	1
2	HgCl_2_	30.68 (± 0.29)	0.06
3	Pd	20.55 (± 0.34)	0.04
4	Ni	14.82 (± 0.15)	0.03
5	Ar	21.65 (± 0.33)	0.04
6	Cr	25.17 (± 0.27)	0.05
7	1,4 Dioxane (1,4 D)	27.39 (± 0.73)	0.06
8	2,4-Dichlorophenol (2,4 DCP)	13.22 (± 0.16)	0.02
9	4-Octylphenol (OP)	24.79 (± 0.24)	0.05
10	Nonylphenol (NP)	11.01 (± 0.18)	0.02
11	Bisphenol A (BPA)	27.99 (± 0.20)	0.06

### Real sample analysis

According to reports, picloram is highly versatile in soil and may readily leak into waters or spread from plants treated with it through the roots to neighboring non-targeted crops^9^. The practical application for picloram detection using the nanoprobe UZMWCNT+rGO/AuNPs/GCE in real sample matrices such as soil and rice water was evaluated by SWV using the spike and recovery method. It is worth mentioning that both the samples require no pre-treatment except for filtration using a Whatman filter paper 1. For the analysis, we have spiked varying concentrations of picloram in 0.1 M PBS buffer equilibrated rice water sample followed by a response study via SWV. The analysis of soil sample was also carried out in a similar way. The outcomes of the experiments were analyzed in terms of % recoveries and thus were calculated via Eq. (4)

4 $$\% {\text{ Recovery}} = \frac{{\left[ {\text{S}} \right]picloram - \left[ {\text{B}} \right]picloram{ }}}{{\left[ {{\text{SS}}} \right]picloram}} \times 100$$

where [S]_picloram_ and [B]_picloram_ are the analytical responses of the nanoprobe in real samples spiked with picloram and blank, respectively, and [SS]_picloram_ is the analytical response of same concentration picloram in PBS solution.

**Figure 8:**
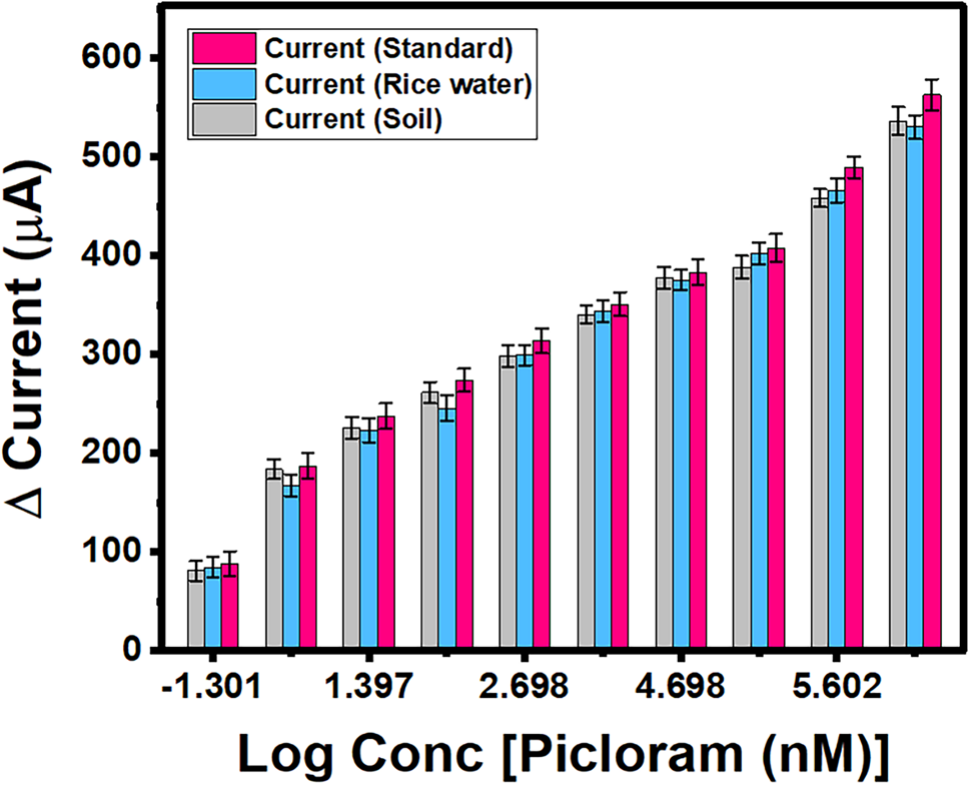
Real sample analysis of the UZMWCNT + rGO/AuNPs/GCE sensor in rice water and soil.

The SWV responses for varying concentrations of rice water (blue) and soil (grey) are represented in a histogram depicted in Fig. 8. An increased concentration of picloram spiked in rice water resulted in an increase in the current responses and the calculated recovery % for picloram in rice water was found to be 88.58% - 96.70% (RSD <3.5%, n=3). The linear increment in the SWV responses was represented mathematically as ΔI (μA) = 154.24(±11.63) + 51.77(±3.12) Log conc [Picloram(nM)] with a correlation coefficient of 0.97. The sensor probe UZMWCNT+rGO/AuNPs/GCE was also evaluated for detection of picloram spiked in the soil sample. An increased concentration of picloram spiked in the soil also resulted in an increase in the current responses and the calculated recovery % was found to be 89.57% – 93.24% (RSD <3.5%, n=3). Similarly, the SWV responses were recorded and a mathematical representation of ΔI (μA) = 160.16(±12.81) + 50.18(±3.43) Log conc [Picloram (nM)] was obtained with a correlation coefficient of 0.96 when compared with the standard calibration curve (pink). After conducting the study, it can be noted that the overall response of the constructed sensor was highly selective for picloram. Throughout the real sample study, no foreign peaks were observed in this experiment thus, indicating that the several unknown molecules/constituents that are present in the real sample matrices do not interfere with the designed sensor probe in our experimental settings.

### Reproducibility and stability studies

**Figure 9:**
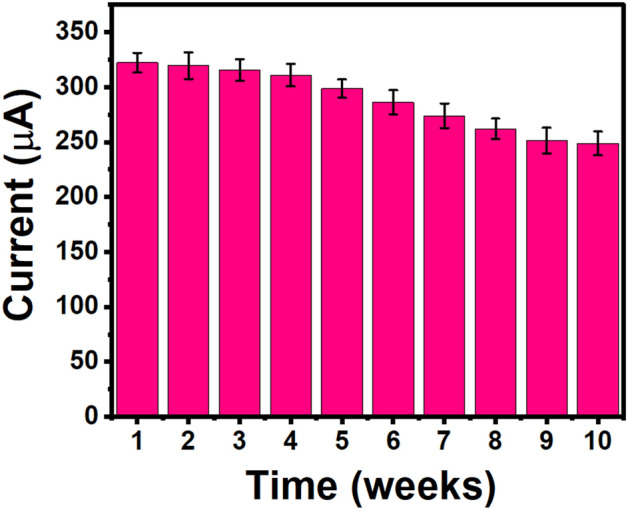
Histogram showing durability and electrode stability of the sensor probe over a period of ten weeks.

The other factor that determines whether any such sensors are commercially viable is their reproducibility and precision in analysis^59^. To evaluate such parameters of the constructed UZMWCNT+rGO/AuNPs/GCE sensor, the current responses were obtained at three individually constructed sensors. When the same manufacturing technique was used, the resultant current values were observed to be trivial with an error % of <4.8. Thus, this demonstrated that the designed sensor is highly reproducible and stable. Additionally, the developed sensor probe UZMWCNT+rGO/AuNPs/GCE has also been evaluated for its long-term stability. Following that, it was observed that the current response lessens with time, indicating that the created sensor is stable for up to ten weeks. (Fig. 9). The stability and slight variations in chip-to-chip analysis were possibly because of slight variations in the probe fabrication and/or a few handling flaws.

## Conclusion

In summary, we described the use of a simple, rapid, and cost-effective sensor system that requires no biological recognition element for the precise detection of trace levels of the herbicide picloram. In this work, we have experimentally demonstrated that the conversion of MWCNT to UZMWCNT leads to an enhanced electrical and catalytic properties. The nanohybrid synthesized in this work was found to be highly catalytic and efficient in facilitating the electrooxidation of picloram at the fabricated electrode surface. The system was found to be highly accurate and sensitive with a wide linear range of 5 × 10^-2^ nM to 6 × 10^5^ nM, low LOD of 2.31±0.02 (RSD <4.1%) pM, LOQ of 7.63±0.03 pM and a quick response time of 0.2s. Based on this study, most of the possible interferents do not affect the electro-oxidation output of picloram, even in a concentration of more than 50-fold, thus proving that the suggested approach is highly selective. The approach was subsequently effective for the detection of picloram in soil and rice water samples. In addition, our work requires no sophisticated sample pre-treatment methods and shows good recovery percentages from soil and rice water samples in the range of 88-97%. Finally, the strategy described in this work has several features such as simplicity and ease of manufacturing, highly stable, rapid, and cost-effective, and requires no bio-recognition element. In the future, this system can be made deployable for field testing since the detection range falls under the permissible EPA-approved limit. This suggests the ability and direct application of the fabricated sensor in the field for on-site surveillance of picloram.

## Data Availability

The datasets used or analyzed during the current study are available from the corresponding author on reasonable request.
